# Utilization of Recycled Brick Powder as Supplementary Cementitious Materials—A Comprehensive Review

**DOI:** 10.3390/ma17030637

**Published:** 2024-01-28

**Authors:** Özlem Sallı Bideci, Alper Bideci, Ashraf Ashour

**Affiliations:** 1 Faculty of Art, Design and Architecture, University of Düzce, Düzce 81600, Türkiye; ozlembideci@duzce.edu.tr (Ö.S.B.); alperbideci@duzce.edu.tr (A.B.); 2 Faculty of Engineering and Digital Technologies, University of Bradford, Bradford BD7 1DP, UK

**Keywords:** waste management, supplementary cementitious material (SCM), waste brick powder (WBP), particle size, pozzolanic activity

## Abstract

Over the past two decades, extensive research has been conducted to explore alternative supplementary cementitious materials (SCMs) in order to address the environmental concerns associated with the cement industry. Bricks, which are frequently preferred in the construction sector, generate a lot of waste during the production and demolition of existing buildings, requiring environmentally sustainable recycling practices. Therefore, many studies have been carried out in recent years on the use of brick waste as supplementary cementitious materials (SCMs) in cement mortar and concrete production. This critical review evaluates the impact of waste brick powder (WBP) on the mechanical and durability properties of mortar and concrete when used as a partial replacement for cement. It was observed that the properties of WBP-blended cement mortar or concrete depend on several factors, including WBP particle size, replacement ratio, pozzolanic activity, and mineralogical structure. The findings indicate that WBP with a particle size range of 100 µm to 25 µm, with a maximum cement replacement level of 10–20%, exhibits a positive impact on the compressive strength of both mortars and concretes. However, it is crucial to emphasize that a minimum curing duration of 28 days is imperative to facilitate the development of a pozzolanic reaction. This temporal requirement plays a vital role in realizing the optimal benefits of utilizing waste brick powder as a supplementary cementitious material in mortars and concretes.

## 1. Introduction

Concrete is one of the main material in construction due to its durability, flexibility, and wide availability. Additionally, the production of cement, the main binder of concrete, accounts for approximately 7% of global CO_2_ emissions [1]. CO_2_ intensity in cement production has increased by about 1.5% annually between the years 2015 and 2021. In contrast, annual reductions of 3% by 2030 are required to move towards a net zero emissions scenario by 2050. To promote the sustainable development of the cement industry, it is noted that there is a need to focus on two key areas, namely, reducing the clinker-to-cement ratio (including greater uptake of blended cement) and using innovative technologies such as carbon capture, storage, and clinkers made from alternative raw materials [2]. However, the Global Cement and Concrete Association (GCCA) proposed a seven-point plan to reduce emissions by a further 25% over the next decade, identifying the replacement of clinker, which is the main component of Portland cement, with additional materials such as fly ash (a by-product of the energy sector), ground granulated blast furnace slag, calcined clays, unburned and ground limestone or recycled concrete fines as a key priority, and the use of reprocessed and recycled materials will increase through efficient use of resources and products [1]. Many studies have looked into the availability, efficacy, and efficiency of waste materials with pozzolanic properties as a substitute for cement. The necessary components ought to be a byproduct of an original source with high silicon (Si) and aluminum (Al) content [3]. SCMs are widely used in concrete mixes by replacing part of clinker in cement or replacing part of cement in concrete [4,5]. Therefore, in the case of replacing parts of cement in concrete with SCMs or clinker being partially replaced with SCMs, making blended cement is a solution to reduce CO_2_ emissions from the cement and concrete industry [6]. This solution is the fastest short-term solution for the reduction of CO_2_ emissions [7].

Most research studies have shown that replacing ordinary Portland cement (OPC) or part of it in concrete with SCMs reduces the amount of cement and the carbon emissions associated with cement production [8,9,10,11,12]. Moreover, research has shown that SCMs have both pozzolanic [13,14] and filler properties, which are embodied in their ability to improve the mechanical and durability properties of concrete [10,15,16,17,18]. Ongoing research is exploring various alternative waste materials as potential SCMs instead of the well-known ones such as fly ash, ground granulated blast furnace slag, and silica fume. Several materials that are being investigated include waste glass [19], ceramic ETP sludge waste [20], gravel wash mud [21], solidified wastewater treatment sludge [22], rice husk ash (RHA), liquid crystal display [23], and waste brick [24].

Waste bricks are plentiful and are produced as a result of faulty production or construction and demolition activities [25], requiring proper disposal from an environmental perspective. In the literature, there are many studies on the use of waste bricks in cementitious systems as a cement substitution [26,27], a partial replacement of cement and fine aggregate [28], a fine and coarse aggregate [29], a fine aggregate [30,31], and a coarse aggregate [32,33]. The use of WBP is of great importance [34], especially because it saves a significant amount of energy and primary raw materials each year, extends the life of the landfill, requires less clinker production, has favorable mechanical and durability properties in concrete, reduces the amount of CO_2_, NOx, and other air pollutants in cement production, and offers many alternative uses for recycled brick-based products without compromising cost and quality. 

This paper reviews recent research studies that investigate the effect of replacing cement with WBP on the mechanical and durability properties of cementitious materials. It discusses the properties of WBP used as SCM that affect the performance of mortar and concrete, examines the latest data on mechanical and durability properties, and highlights recommendations for future research on the use of WBP based on identified needs.

## 2. Properties of WBP as SCM in Mortar and Concrete

In many studies, WBP was used as an SCM, replacing cement in the production of mortars and concrete [34,35,36,37,38,39,40]. In mixtures where waste concrete and brick powder are used together, the replacement ratios and particle size of powders are important when evaluating the fluidity and rheology of the mixture [41]. Thus, the use of ground WBP as a partial replacement of cement in concrete and mortar significantly affects the pozzolanic reaction and mechanical properties. The effects of WBP content, particle size and curing age on the properties of mortar and concrete mixtures are discussed under the following headings listed below. 

### 2.1. Effect of WBP Particle Size and Curing Period on Compressive Strength

WBP used in the development of artificial pozzolans must meet certain requirements. In addition to containing a minimum of amorphous silica and alumina to ensure chemical reactivity, WBP needs to be of at least the same fineness as Portland cement so that its chemical and physical effects can be improved [42]. In cement-based material containing WBP with various particle sizes, pozzolanic activity may increase with decreasing particle size [43]. In hybrid mixtures containing concrete and brick powder, the strength development of the blended mortar is improved by increasing the WBP ratio or decreasing the particle size [41]. 

**Figure 1:**
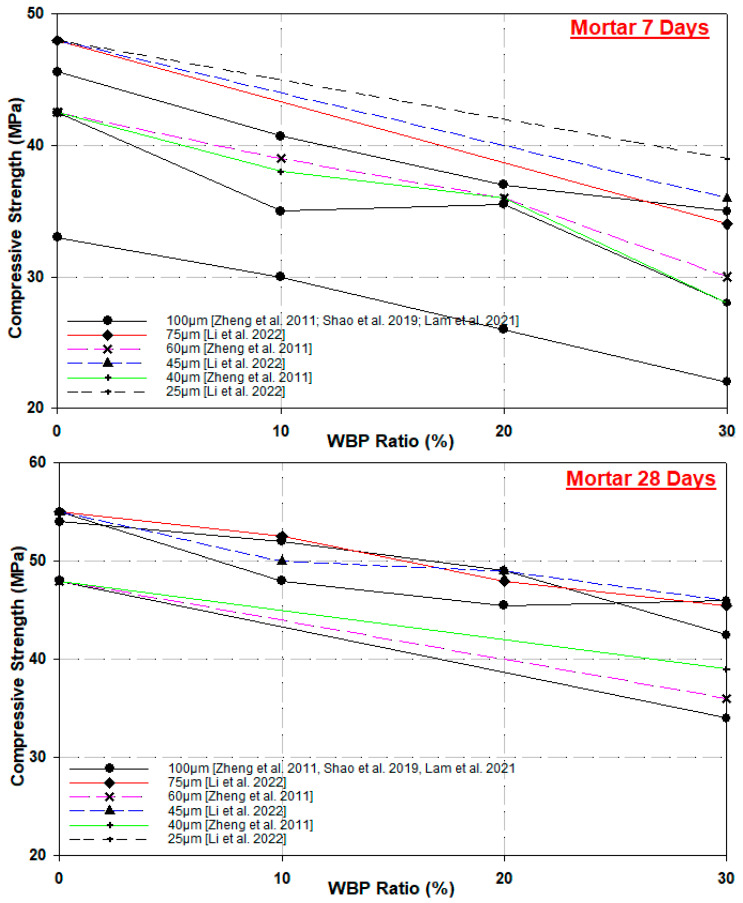

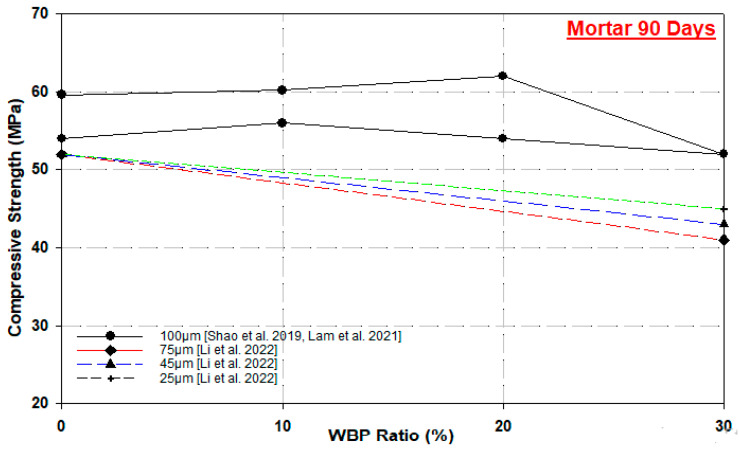
Compressive strength, particle size, and replacement ratio of WBP in mortars [38,44,45,46].

The particle size and substitution ratios of WBP have an influence on the properties of mortars and concretes. The use of WBP with different particle sizes and substitution ratios was examined in two groups under the headings of mortar and concrete. The use of WBP in mortar samples was investigated at 0%, 10%, 20%, and 30% substitution rates and particle sizes of 100 μm, 75 μm, 60 μm, 45 μm, 40 μm, and 25 μm as observed in the literature. The particle size-compressive strength graphs of WBP-substituted mortars for 7, 28, and 90 days are given in Figure 1.

As seen in Figure 1, among the 28-day WBP-substituted samples with 75 µm, 45 µm, and 25 µm particle sizes, the highest compressive strength was obtained from the samples produced with 25 µm particle size and 30% additive ratio; thus, the relationship between particle size and additive ratio could be clearly observed. The compressive strength ratio of mortars gradually increases with the increase in the curing age, showing better strength development than the reference mortar. Therefore, reducing the particle size of WBP would be a feasible way to improve the mechanical properties [44]. The compressive strengths of 28-day mortars with 40 µm- and 60 µm-particle-size WBP-substituted samples compared to the reference mortar indicate that the highest compressive strength value was obtained by the 10% blended samples with 40 µm particle size. The compressive strength of WBP-substituted specimens with a particle size of 60 µm showed a similar trend and the compressive strength decreased with the additive ratio. As the average particle size of WBP increases, the compressive strength decreases at the same substitution ratio. Hence, it is clearly seen that the effect of WBP on compressive strength depends on the substitution level and average particle size [38]. In the other two studies [45,46], it is seen that the compressive strengths of WBP-substituted specimens were lower than the reference specimens in all age groups for specimens with a particle size of 100 µm. The highest compressive strengths for 90-day specimens were 56 MPa and 62.2 Mpa, these substituted specimens which are 10% and 20% respectively increased 3.7% and 4.03% specimens. Moreover, 30% of substituted specimens were determined to be 52 Mpa, with 3.7% and 13% decreases compared to the reference specimen. The contribution of WBP to strength at early ages is not as good as cement. The strength of mortars decreases with increasing WBP content, especially at early ages, and gradually improves with curing time [45,46].

**Figure 2:**
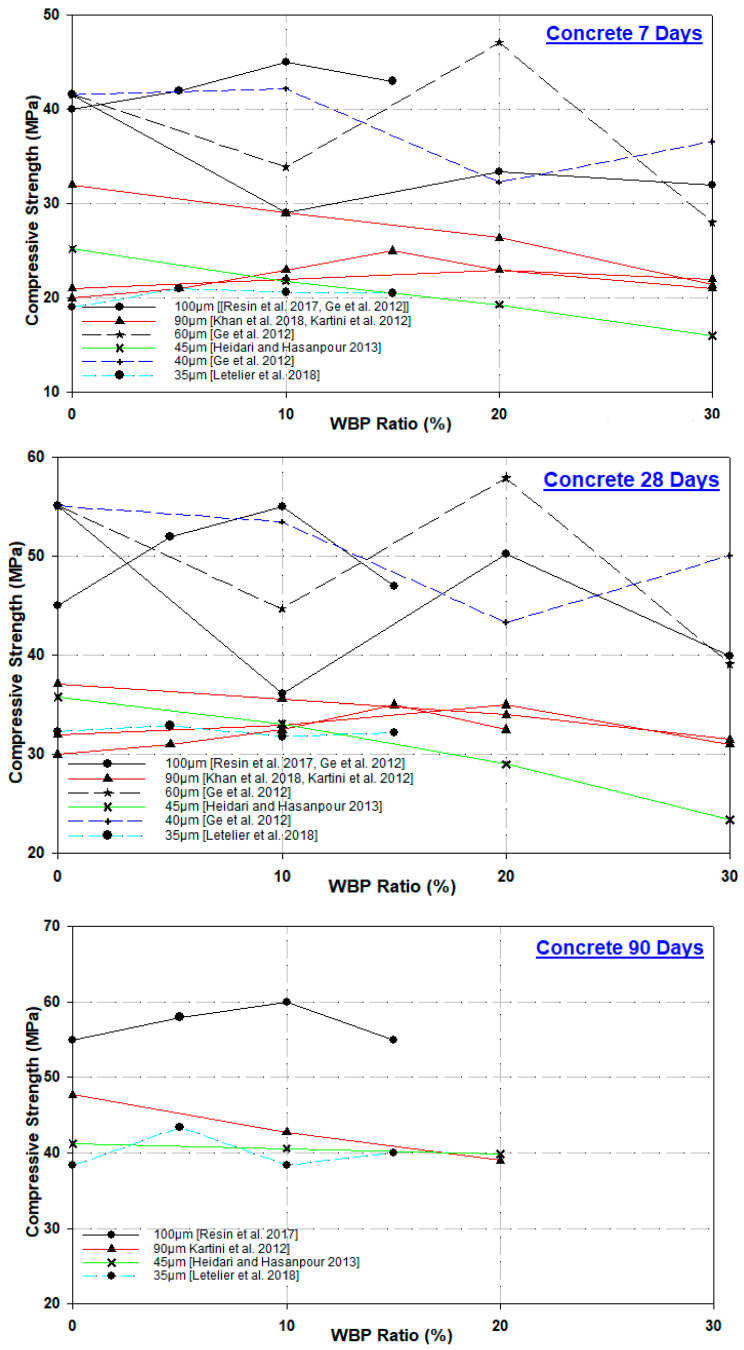
Compressive strength, particle size, and replacement ratio of WBP in concretes [26,34,36,47,48,49].

The use of WBP in concrete specimens was also investigated at different substitution rates (0%, 10%, 20%, and 30%) and different particle sizes (100 μm, 75 μm, 60 μm, 45 μm, 40 μm, and 35 μm) in the literature. The WBP particle size and replacement ratio against compressive strength plots at 7, 28, and 90 days are presented in Figure 2.

Figure 2 shows that the highest 28-day compressive strength of specimens produced with 0%, 5%, 10%, and 15% WBP admixture ratios, under-35 μm particle size, was obtained from 5%-WBP-substituted concrete with 32.93 MPa. The 90-day compressive strength results of 43.4 MPa, 38.4 MPa, and 40.0 MPa were higher than the reference concrete (38.4 MPa) at all admixture rates. According to the results, WBP substitution up to 15% provides a dense structure in concrete and causes a filling effect with pozzolanic properties [26].

The proportion of water to cementitious material is a crucial factor in determining the mechanical characteristics of concrete that includes WBP, much like conventional concrete. Therefore, the addition of recycled brick powder reduces the compressive strength of concrete, and with proper mix design, the compressive strength can reach 50 MPa or higher than the reference specimen. Ge et al. [47] concluded that a water/cemented material ratio of 0.26, sand ratio of 33%, brick-powder with a particle size of 0.06 mm, and 25% cement substitution would give optimal compressive strength. Heidari and Hasanpour [34] found that the compressive strengths of 28-day specimens produced with 10%, 20%, and 30% WBP substitution at a particle size of 45 μm decreased by 7.5%, 19.0%, and 34.6%, respectively, compared to the reference concrete, while this decrease was 1.4%, 3.2%, and 10.3%, respectively, in 90-day specimens. According to the values obtained, as the WBP substitution ratio increases, the compressive strength of the specimens decreases at early ages and loses strength at later ages [34]. When the 7- and 28-day samples with smaller than 90 µm particle size were examined, the compressive strength of the samples with 20% substitution increased by 10% compared to the reference sample in both age groups, while the 28-day samples with 30% substitution decreased by 3% compared to the reference sample [50]. In another study with WBP substitution with particle size smaller than 90 µm, the compressive strength of the specimens did not exceed the strength of the reference concrete. However, the compressive strength values of the specimens were still above the 30 MPa compressive strength value targeted by the researchers [48]. Specimens produced with 15% WBP cement substitution under 90 µm particle size showed maximum strength at 7 and 28 days of curing time. The specimens with 20% substitution increased by 15% and 8%, respectively, compared to the reference specimens. The pozzolanic effect of WBP resulted in the production of more C-S-H gels, and the filling effect of the finer particles in the concrete caused an increase in strength [27,49]. In WBP-cement-substituted specimens below 100 µm particle size, specimens with a 10% substitution rate gave the highest compressive strength in all age groups. The specimens with a 15% substitution ratio showed an increase of 7.5%, 4.4%, and 0.0% in all age groups, respectively, compared to the reference specimens. As in other studies, WBP, together with the pozzolanic reaction, effectively fills the pores and causes a dense micro concrete structure, leading to an increase in strength [36]. Therefore, WBP below 0.15 mm can play the role of micro-filling and reach a denser state, resulting in an increase in the compressive strength of the mortar [30]. The main role of WBP particles in early hydration is their filling effect due to their physical properties rather than chemical activities [51]. However, the chemical composition of WBP meets the standard requirements as a pozzolanic material, and its 10% substitution ratio with cement provides a significant improvement in the compressive strength of concrete. Despite the increase in compressive strength alone, the increase in the WBP ratio causes a decrease in strength. For this reason, it is recommended to use no more than 15% WBP for concrete production [35,52].

When mortar and concrete samples in the above studies are generally examined, the particle size of WBP has been one of the factors affecting the pozzolanic activity. Increasing the WBP replacement ratio and decreasing the particle size increased the pozzolanic activity and contributed positively to the strength development. In general, it can be observed that the early age strength decreases with the increase in WBP ratio, but concrete and mortar exhibit little strength loss with curing time. While studies focused on grain size and the substitution ratio, they also emphasized that attention should be paid to the water/cement ratio. It can be concluded that the particle size below 100 µm and 25 µm and 10–20% cement substitution has a positive contribution on the compressive strength of concrete or mortars. 

Curing age can also significantly affect the strength gain of concrete [38,45,46,53]. The occurrence of C-H content in the reference specimens during the curing process in WBP-substituted pastes was significantly higher than that in WBP-containing pastes. The decrease in C-H content is attributed to the interaction between the reactive pozzolanic phases in WBP-containing paste specimens and C-H released during cement hydration [54]. Generally, the lower early age strengths are due to the fact that the specimen microstructure becomes less dense by increasing its porosity in the early stage due to the increase in the substitution rate since the specimen does not participate in the cement hydration reaction and no pozzolanic reaction occurs [40,45,55,56,57]. Also, curing temperature has an effect on the hydration process of cement. High temperature increases the hydration of cement, and this effect becomes more evident as the temperature increases. With increasing temperature, the cumulative heat of the pastes also increases. When the curing temperature is 60 °C, the pozzolanic reaction of RBP can affect the release of heat of hydration during the slowdown and stable period [58]. Furthermore, the water absorption capacity of WBP reduces the effective water/cement ratio in the samples, and the reduction of water content may weaken the hydration reaction of cement. Therefore, the more WBP is substituted, the more pronounced the reduction effect will be in the hydration products [28]. However, after 28 days of hydration, an appropriate amount of WBP plays a role in refining the pore size and improving the pore structure [57]. The strength increase in the samples containing WBP is due to the higher amorphous silica content than the reference samples in terms of the formation of the pozzolanic reaction. The amorphous silica content, which is necessary for the pozzolanic reaction to occur, and the additional C-S-H gel formation leads to better strength by improving the pores [59].

A general review of the above studies shows that limiting WBP as a cement substitute in cement-based concrete to less than 20% would be appropriate for optimum compressive performance. The amorphous silica content in pozzolana increases pozzolanic activity. At the end of the pozzolanic reaction, the amount of Ca(OH)_2_ decreases, the paste becomes denser due to the formation of C-S-H gels, and the improvement of the gaps between the cement particles leads to an increase in strength. Thus, it can be concluded that the pozzolanic reaction, which reduces the amount of Ca(OH)_2_ and improves densification, increases the strength of the specimens after 28 days according to the curing age of the specimens, and it can also be concluded that the strength decreases when cement is substituted by WBP at a higher rate.

### 2.2. Pozzolanic Activity and Mineralogical Structure 

The pozzolanic activity of WBP is particularly dependent on the amorphous phase content, particle size distribution, and specific surface area [60]. According to the ASTM C618 [61] strength activity index approach, in order for the material to have pozzolanic properties, the sum of Si_2_O, Al_2_O_3_, and Fe_2_O_3_ should be at least 70% in its chemical composition. Aliabdo et al. [62] detected the presence of portlandite, ettringite, calcite, quartz, and C-S-H in the diffraction peaks of all paste samples in which WBP was substituted into the cement paste. It was also stated that WBP substitution up to 25% had little effect on the mineral compositions of the cement matrix. Furthermore, it was indicated that the WBP used was in accordance with the strength activity index approach outlined in ASTM C618 and summarized in ASTM C311 [63], which limits oxides to at least 70%. In another study, in WBP-substituted mortar with an amorphous content of 35% and a parameter supporting pozzolanic activity (Si_2_O + Al_2_O_3_ + Fe_2_O_3_ ≥ 70), despite a significant decrease in strength values compared to control specimens at early ages (3 and 7 days) with substituted specimens, strength values above 50 MPa were obtained after 365 days of curing [64]. The chemical compositions of WBP used in the studies are given in Table 1. When Table 1 is examined, it is determined that the total of SiO_2_, Al_2_O_3_, and Fe_2_O_3_ required for the pozzolanic property of the WBPs used in the studies is over 70%.

Ortega et al. [71] stated that 10%- and 20%-WBP-substituted mortars, which are formed by the pozzolanic process with additional C-S-H phases, show longer life and better service properties compared to the reference mortars; however, the hydration of clinker and the presence of portlandite are needed for the development of pozzolanic varieties. Since the clinker content of the substituted samples is lower than the 10%-substituted ones, less portlandite configurations were made to the 20%-substituted parts at the same curing ages. When the substitution rate is over 30%, WBP substitution results in an increase in quartz content and a significant decrease in C-H and calcite content [82]. 

For pozzolanic activity, amorphous phases formed by a certain amount of aluminosilicates in the combustion process of clay minerals are required [58,69]. It was determined that quartz was the main mineral phase in addition to a small amount of feldspar and hematite in WBP, and the amount of amorphous phase was 19.6% in the hydration product analysis of the samples via X-Ray diffraction (XRD). Similarly, Ortega et al. [71] identified inorganic crystalline phases, silica (sand used to adjust the plasticity of the brick green mixture), illite (the main mineralogical component of clay used in brick production), and hematite (used to reduce the firing temperature and favor the formation of liquid phases).

**Figure 3:**
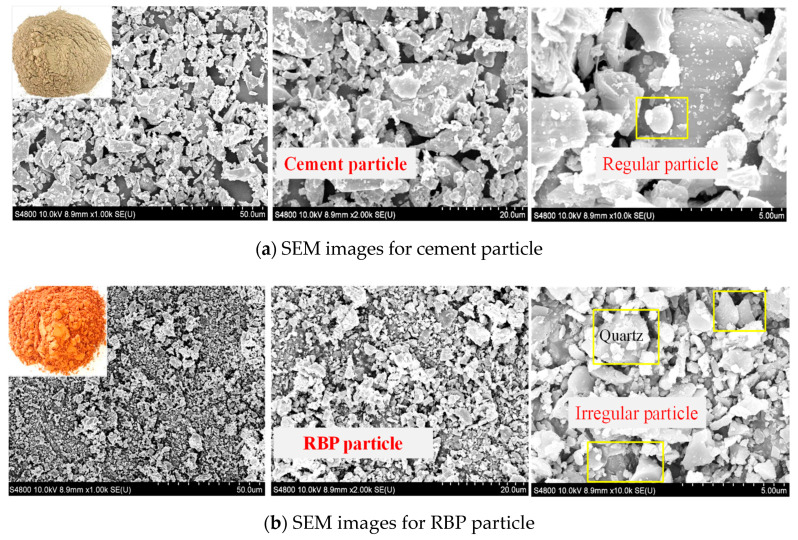
Microstructure of cement and recycled WBP [58].

Based on SEM-EDS analysis of 28-day-old specimens, the spherical particles were identified as C-(A)-S-H gels, and it was observed that the number of networks and fibrous C-S-H gels was less, and the number of spherical particles was more for the specimens with 20% and 40% WBP substitution than the reference specimen. Thus, the inclusion of WBP promotes the formation of spherical particles, making the structure compact and increasing strength development [58]. Similarly, Zhao et al. [43] stated that WBP particles tend to be refined and spherical, which will increase the specific surface area and pozzolanic activity of WBP. The WBP grain has a semi-oval and semi-smooth surface shape and is composed of morphologically irregular particles of quartz and feldspar, which are necessary components for pozzolanic activity [62,83]. Moreover, its microstructure is more irregular than a cement particle, with more edges and corners [30,71,82]. In SEM analysis of WBP, it was generally observed that the main crystalline phase contains quartz and mineral compositions of albite, calcite, anorthite, and sanidine [28,30]. An example of the microstructure of cement and recycled WBP is given in Figure 3.

## 3. Durability Properties

### 3.1. Water Absorption 

**Figure 4:**
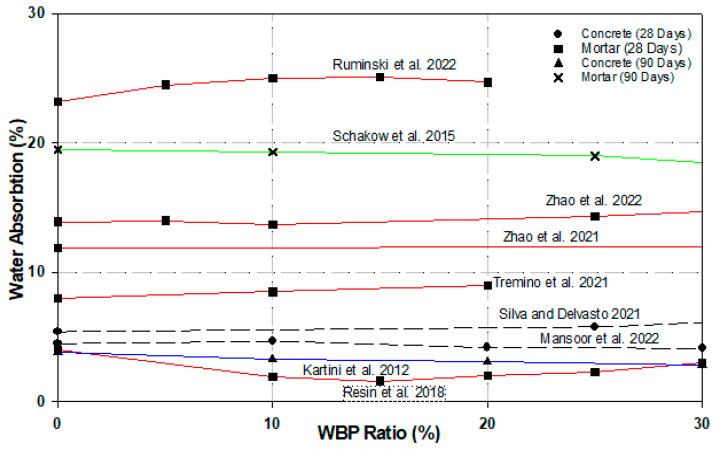
Water absorption values of WBP-admixed mortar and concrete (%) [24,36,48,73,77,85,86,87,88].

The pore characteristics in concrete is evaluated by measuring the sorptivity, water absorption, and water permeability rate of unsaturated specimens by immersing them in water with or without a water head pore [84]. Test results of water absorption rates of WBP-substituted mortars and concretes at the end of 28, 90, 150, and 240 days curing period are presented in Figure 4. In Figure 4, When the study of Zhao et al. [85] on the water absorption of mortars is evaluated in Figure 4, (in the 28-day samples) it is seen that the water absorption value decreases in the 10% substituted samples compared to the (0%) water absorption value of the reference mortar and that the water absorption value increases in the 5%, 25% and 50% substituted samples [85]. In a similar study, it was observed that in the samples produced with 0%, 50%, and 100% WBP substitution rates, the water absorption values of the samples with 50% and 100% substitution rates increased compared to the reference mortar water absorption values [73]. Moreover, 10%- and 20%-WBP-substituted mortars showed an increase in water absorption rate similar to other studies [24,77]. Water conductivity decreases especially with a 15% WBP substitution of cement. This decrease in permeability properties is due to the pozzolanic activity of WBP, and an additional hydration gel is formed by hydroxide and pozzolanic reaction products that fills the pores of WBP [86]. The water absorption percentages of mortars with 0%, 10%, 25%, and 40% substitution rates decreased by 1.03%, 2.56%, and 9.7%, respectively, after 90 days of curing. Water absorption values were below the control mortar values at each ratio with WBP substitution. This indicates that the mortar without WBP has a more porous structure than the mortar with WBP. The density of the cured mortars appears to be due to both the physical and pozzolanic effect of the addition of WBP by reducing the volume of pores in the hardened matrix [87]. 

In 10%-WBP-substituted concretes, the water absorption rate decreased by 26.3% and 15.0% at 150 and 240 days of curing age compared to the reference concrete [36]. Furthermore, 10–20% WBP substitution with cement may be related to the improvement in pore structure by reducing water absorption [89]. In 25%- and 50%-substituted concretes, the water absorption rates of the samples increased by 7.04% and 35.74%, respectively, compared to the control sample at the end of the 28-day curing period. The decrease in water absorption rate from 28 days to 360 days in 25%- and 50%-substituted concretes for the control sample was 36.85%, 47.75%, and 58.79%, respectively. It was observed that the water absorption values of concrete specimens increased at the end of 28 days of curing time, while the water absorption values decreased at the end of 360 days of curing time due to the decrease in pore size in the matrix due to hydration and the pozzolanic reaction [90]. Moreover, 30% WBP substitution was reported to be the most effective ratio in reducing the water absorption of concrete. With curing periods of 28, 60, 90, and 120 days, water absorption rates decreased with increasing WBP cement substitution according to age. The fine brick powder filling the space between the coarse cement particles ensures the compactness of the concrete and subsequently reduces the degree of water absorption [49]. In concrete specimens where 5% burnt clay brick powder blended with rubber was used, 5% substitution with cement resulted in a 16.7%, 22.7%, and 33.3% reduction in water absorption compared to the control concrete for concrete classes of 20 MPa, 25 MPa, and 30 MPa, respectively [91]. Due to its high pozzolanic activity and good micro-aggregate filling effect, WBP milled below 20% improves the water transport performance of cementitious composites by improving the pore structure [41]. 

In general, the use of mineral admixtures, including WBP, reduces the water absorption of mortar mixtures. This phenomenon can be explained by the physicochemical effect of mineral admixtures in two different ways. Chemically, as previously emphasized, as a result of the pozzolanic reaction (conversion of C-H to C-S-H), the existing pores in the matrix are reduced, making it difficult for water to enter the mixture. Thus, the permeability property of the mixture is positively affected. Physically, since mineral admixture has a higher fineness than cement, when used instead of cement, it physically closes the pores and improves the permeability of the mortar mixture [92]. In most of the studies reviewed, it is understood that WBP substituted with cement at a certain content ratio and curing time improves the pore structure in the transition zone of mortars and concretes, increases the compactness of the mortar, and contributes positively to the water absorption resistance.

### 3.2. Drying Shrinkage

Drying shrinkage is an intrinsic phenomenon caused by the change in moisture content to which an inorganic binder is subjected and manifests as volume shrinkage [93]. Drying shrinkage can lead to a high probability of cracking in cementitious materials due to moisture transport, resulting in poor long-term performance and service [94]. For this reason, it is important to know the effect of WBP content on the drying shrinkage of cementitious materials. Lam et al. [46] used mortars used with waste clay brick dust at a rate of 10%, 20%, 30%, and 40% by weight in their studies for 7 and 28 days, respectively, according to the drying shrinkage value of the reference sample; they stated that drying shrinkage decreased with the increase in WBP ratio in the mixtures, with a decrease of 26% and 24% in samples with 10% additive, 40% and 33% in samples with 20% additives, 46% and 43% in samples with 30% additives, and 53% and 48% in samples with 40% additives. The hydration of WBP is lower than that of cement, which reduces the hydration products with WBP substitution. In addition, capillary pores can be improved by the pozzolanic activity of WBP, leading to lower shrinkage [46]. Wu et al. [28] determined that the drying shrinkage of mortars with 10%, 30%, and 50% WBP substitution was 9.5%, 15.9%, and 2.7% lower than that of the normal mortar due to the added WBP improving the pore structure of the cementitious materials. In their study, mortar containing 30% WBP had the lowest drying shrinkage. Silva et al. [88] observed that the 56th-day reference mortar shrinkage was about 54% higher than that of mortars with 50% additional cement, indicating that mortars with cementitious material (masonry debris) provided a lower drying shrinkage at an early age, suggesting that the addition of recycled waste powder consisting of clay brick waste and old hardened cement paste as a cement substitute improve the shrinkage resistance. Similarly, it was reported that the inclusion of recycled material in the mix significantly reduces shrinkage at all ages [95]. Drying shrinkage increases rapidly at early ages and slows down at later ages [46]. The presence of additional cementitious materials can help to control hydration and moisture loss at an early age and reduce drying shrinkage by producing additional C-S-H gel through pozzolanic reactions at later ages [94].

### 3.3. Resistance to Chloride Attack

**Figure 5:**
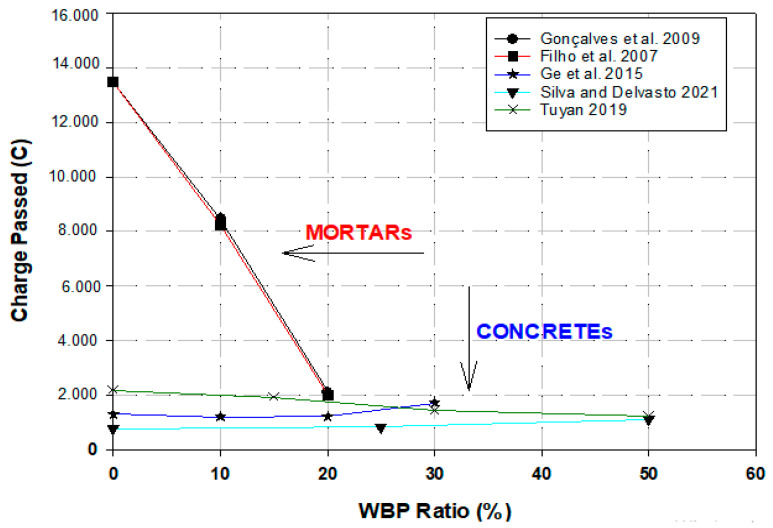
Chloride ion penetration values of mortar and concrete samples with different WBP substitution rates [56,88,89,97,99].

The chlorine effect differs from other adverse conditions to which concrete is exposed in that, instead of affecting the concrete directly, it causes corrosion of the steel reinforcement in concrete. In essence, the deterioration of concrete under the influence of chlorine is the formation of cracks in the concrete surrounding the steel reinforcement because of the expansion of its volume due to the corrosion of the reinforcement alone. Corrosion starts when the passive layer around the steel reinforcement, which forms spontaneously and immediately after the start of cement hydration and prevents corrosion, breaks down under the influence of chlorine entering concrete [96]. Tremino et al. [24] reported that the effect of brick powder was examined at relatively short curing ages since the required service life of real structural elements is generally long. Furthermore, it was stated that it would be correct to characterize the effect in the long term to assess whether new additions such as brick powder and other pozzolanic materials are sufficient for use in real structural elements. Accordingly, in their study, they observed that the performance of mortars produced with clinker substitution of WBP compared to control mortars, especially pore structure and chloride diffusion after four years (1500 days), generally improved due to the pozzolanic activity and filling effect of WBP. Figure 5 shows the chloride ion penetration values of mortar and concrete samples with different WBP substitution ratios.

Figure 5 shows that for mortars with 10% and 20% WBP substitution, the chloride ion penetration value decreased significantly compared to the control sample, i.e., from 13,500 Coulomb (C) to 8250 C and 2000 C, respectively. This may be related to the pore size improvement of WBP material [89]. Moreover, it is seen that the use of ground calcined clay bricks at 10% and 20% substitution rates in mortars decreased the chloride ion penetration of the control sample by 1.5–6 times from 13,487 C to 8460 C and 2111 C for 10% and 20%, respectively. The use of calcined clay promotes an overall reduction in the electrical charge passing through the specimen [97]. Ortega et al. [71] stated that the forced chloride migration test of 10% and 20% WBP substitution in cement was lower than that of CEM I mortars, and this favorable performance may be due to both the pozzolanic activity and the filling effect of the powder. In the experiment with 10%, 20%, and 30% WBP substitution in concretes, 30%-WBP-substituted concrete was determined to have the lowest resistance to chloride ion penetration. The total passing load was between 1196 C and 1742 C [56], indicating that all specimens had low chloride ion penetration according to ASTM C1202-97 [98]. Although the chloride penetration resistance of the control concrete was high in concrete specimens with 25% and 50% WBP substitution rate for 180 days, the chloride penetration value was 500 C, 375 C, and 410 C, respectively, compared to the control specimen as an effect of pore size reduction as a result of the pozzolanic reaction of WBP [90]. The chloride penetration values of 15%-, 30%-, and 45%-WBP-substituted concrete were 2181 C, 1922 C, 1427 C, and 1211 C, respectively, compared to the control specimen, and it was determined that there was a decrease in the total passing load [99]. In the study investigating the potential of using approximately 80% WBP and 20% waste concrete powder as the binder material, it was reported that waste powder substitution caused a decrease in chloride permeability, and the concrete with the lowest chloride permeability was obtained from 30%-WBP-substituted samples [100]. 

When the studies are analyzed in general, it is seen that the chlorine permeability of concretes with WBP substitution to cement significantly decreases with age and substitution rate. WBP, with its pozzolanic activity and filling effect, can provide a denser microstructure by changing the hardened cement paste pore structure. Thus, concretes with low permeability can be obtained. It is seen that with 20% WBP substitution in mortar samples and 30% WBP substitution in concrete samples, concretes with values close to the “concrete with low chlorine ion permeability” limit according to the values given in ASTM C1202 standard are produced. These low-chlorine-ion permeabilities obtained indicate that the concretes to be produced with WBP have a much longer service life under the chlorine effect.

### 3.4. Resistance to Sulfate Attack

In hardened cementitious materials, the action of external solutions containing sulfates can lead to intense internal stresses. Sulfates in solution combine with calcium hydroxide (C-H, produced as a result of cement hydration) and alumina (in cement) to produce ettringite. The formation of ettringite can cause expansion and fragmentation, especially in chemical environments [101]. Due to the pozzolanic reaction and pore improvement effect, the substitution of cement with fine brick powder has shown improvement in some durability properties of concrete as well as increased resistance to sulfate attack [59]. Alcharchafche et al. [102] stated that in mortars produced by substituting 5%, 10%, and 15% WBP under sulfate effect (90 days), the strength values increased with the use of 5% WBP due to the higher Al_2_O_3_ and SiO_2_ content, resulting in the formation of C-A-S-H and C-S-H gels, while the use of more WBP caused the strength values to decrease.

In mortars containing red brick powder and ground basaltic pumice, after 36 months of exposure to sulphate attack, deterioration in the form of crumbling and cracking is more pronounced when compared with control cement mortar samples. The substitution improves the sulfate resistance of cement mortars, and the appropriate amount of additive that provides high sulfate resistance is around 15% [103]. The sulfate resistance of heat-treated brick clay mortars with a 20% or 30% substitution rate also shows superior performance compared to the control mortar [104]. Li et al. [105] stated that WBP substitution to cement in mortars can significantly improve the sulfate resistance of the substitution by reducing the strength loss by at least 65%. Filho et al. [89] observed a strength loss of approximately 12% in comparison to control specimens exposed to sulfate solution at 200 days of age, while mortars with ground, calcined WBP showed an average strength loss of 4%. Wild et al. [106] reported that mortars containing brick clay calcined and ground at a temperature higher than 900 °C showed superior sulfate resistance compared to those containing brick clay calcined and ground at temperatures below 900 °C. 

When the studies are analyzed in general, it is seen that WBP substitution to cement is one of the most effective methods by which to provide impermeability to the mortar/concrete produced and to prevent or reduce the damages that may occur.

## 4. Life Cycle Assessment (LCA) 

The recycling of WBP is likely to increase the initial cost of concrete, and their use can only be justified on a life-cycle assessment basis, showing both the cost and environmental benefits. Throughout the life cycle of a product, process, or service, from the stages of raw material acquisition, manufacture, and usage to waste management, life cycle assessment (LCA) is a tool used to evaluate the possible environmental consequences and resources [107,108,109]. Having started as a for comparing the environmental consequences of products, LCA is now a standardized method for establishing a sound scientific basis for environmental sustainability of industry and government [110]. Therefore, the sustainability of WBP and its potential to lessen cement’s negative environmental effects are important factors to consider when using WBP as a cement substitute [111]. LCA, comparing various WBP ingredients to those without WBP (control), is one of the best ways to accomplish this topic.

Nasr et al. [112] have conducted a comprehensive review of the LCA of the use of WBP in concrete. They found that by substituting waste brick powder into cement at different rates (0%, 12.5%, 25%, 37.5%, and 50%), there was a 10.8% to 43.2% reduction in global warming potential. According to a study, the manufacturing of clay brick waste reduced CO_2_ equivalent emissions by 49% to 89% at rates ranging from 18.2% to 71.8% [112]. Fort et al. [70] found that the best mixture made from waste brick dust could save up to 72% in greenhouse gases emitted compared to Portland cement paste, evaluated via a combined assessment of functional and environmental characteristics using the carbon dioxide emission efficiency index. In another investigation by Liang et al. [113], it was confirmed that the addition of WBP improved mechanical performance and decreased current CO_2_ emissions. 

## 5. Conclusions

This paper presents a state-of-the-art review on the substitution of WBP in mortar and concrete. The following main conclusions can be drawn:

- The compressive strength of WBP-substituted mortar and concrete specimens decreases as the WBP admixture ratio increases. Notably, particle sizes ranging from 100 µm to 25 µm and substitution rates of up to 10–20% demonstrate a positive contribution to the compressive strength of the produced samples.
- All WBPs employed in the substitution of cement in mortars and concretes were found to satisfy the criteria supporting pozzolanic activity, specifically with Si_2_O + Al_2_O_3_ + Fe_2_O_3_ ≥70. Additionally, the microstructure of WBP is characterized by irregularity, a rough surface, and an angular structure. The pozzolanic reaction results in the formation of additional C-S-H phases in WBP, thereby contributing to the improvement of pore size in the test specimens.
- WBP contributes positively to water absorption resistance by enhancing the compactness of concrete and mortar at a certain substitution ratio and curing period. A WBP replacement ratio of up to 15% of cement in mortars and 10–20% of cement in concrete was observed to be effective in reducing water absorption. However, WBP improves the pore structure of concrete and mortar, resulting in lower drying shrinkage. Furthermore, WBP substitution significantly enhances the sulfate resistance of mortars and concretes, mitigating strength loss.
- The pozzolanic activity and filling effect of WBP improved the resistance of concrete and mortars to chloride ion penetration. Notably, utilizing 20% WBP substitution in mortar samples and 30% WBP substitution in concrete samples, considering the curing age and substitution ratio, enables the production of concretes with characteristics approaching the threshold for “concrete with low chloride ion permeability” as per the ASTM C1202 standard.
- The utilization of WBP as a substitute for cement in cementitious materials emerges as a promising option by which to mitigate the environmental impact, reducing energy consumption, the emission of CO_2_, and costs, and addressing the challenges associated with solid waste disposal in the construction industry.

In the publications reviewed, factors such as brick firing temperature and grinding process significantly impacted the chemical composition of the powder. To enhance the economic and energy efficiency of construction material production, a detailed investigation into these factors is essential, aiming to determine the optimal ratios in material selection and production. WBP can be further investigated with different particle sizes, water/binder ratios, and replacement ratios to determine the optimum WBP replacement ratio suitable for mortars and concretes.

While existing research studies have comprehensively explored the physical, mechanical, and durability properties of concretes and mortars incorporating WBP, there remains a research gap in investigating the thermal characteristics and acoustic performance of such materials. Additional studies in these areas would contribute to a more comprehensive understanding of the potential applications of WBP in the construction industry.

## Figures and Tables

**Table 1:** Chemical composition of WBP.

Compound	SiO_2_	Al_2_O_3_	Fe_2_O_3_	Si_2_O + Al_2_O_3_ + Fe_2_O_3_ ≥ 70	CaO	K_2_O	Na_2_O	MgO	Loss on Ignition	References
(% by mass)	41.47	39.05	12.73	93.25	0.63	2.81	----	----	----	[24]
	41.47	39.05	12.73	93.25	0.63	2.81	----	----	----	[26]
	76.10	11.80	4.80	92.70	1.30	----	----	1.70	----	[28]
	59.07	13.99	5.90	78.96	12.50	2.72	----	2.14	----	[30]
	64.50	18.40	8.00	90.90	1.90	2.70	1.10	1.50	0.20	[43]
	63.40	25.60	5.45	94.45	0.44	2.78	----	0.36	0.70	[48]
	66.74	18.81	6.03	91.58	1.31	2.66	----	1.69	----	[57]
	54.20	15.40	7.60	77.20	6.80	----	----	2.50	6.20	[62]
	69.99	10.62	4.02	84.63	8.86	2.61	1.02	1.39	----	[65]
	49.90	16.60	6.50	73.00	9.70	4.40	0.50	5.50	2.40	[66]
	57.67	14.91	5.02	77.60	9.81	3.20	1.45	3.74	0.00	[67]
	69.05	23.02	1.52	93.59	2.31	2.59	1.28	1.05	----	[68]
	55.50	17.00	5.80	78.30	10.50	2.80	0.70	2.40	----	[69]
	58.80	19.60	5.70	84.10	6.90	2.90	1.50	2.80	----	[70]
	41.47	39.05	12.73	93.25	0.63	2.81	----	----	----	[71]
	62.01	21.11	7.00	90.12	1.45	3.37	1.60	2.00	----	[72]
	62.80	10.40	16.30	89.50	1.70	2.10	0.60	2.20	0.50	[73]
	71.34	16.21	5.68	93.23	1.51	1.22	0.58	0.73	0.47	[74]
	67.09	17.32	4.75	89.16	4.32	2.91	----	----	----	[75]
	68.15	16.51	7.20	91.86	1.80	----	0.65	0.94	----	[76]
	59.30	14.10	7.60	81.00	2.00	3.40	----	2.30	10.1	[77]
	64.36	8.71	12.86	85.93	2.00	3.05	1.82	----	0.97	[78]
	63.21	16.41	6.05	85.67	---	2.83	1.19	1.11	---	[79]
	51.30	19.30	6.00	76.60	11.50	3.20	1.30	4.50	1.10	[80]
	51.30	20.00	6.00	77.30	11.50	3.20	1.30	4.50	1.10	[81]

## Data Availability

The authors confirm that the data supporting the findings of this study are available within the article.
